# Stimulus-specific adaptation and deviance detection in the inferior colliculus

**DOI:** 10.3389/fncir.2012.00089

**Published:** 2013-01-17

**Authors:** Yaneri A. Ayala, Manuel S. Malmierca

**Affiliations:** ^1^Laboratory for the Neurobiology of Hearing, Auditory Neurophysiology Unit, Institute of Neuroscience of Castilla y León, University of SalamancaSalamanca, Spain; ^2^Department of Cell Biology and Pathology, Faculty of Medicine, University of SalamancaSalamanca, Spain

**Keywords:** auditory, non-lemniscal pathway, frequency deviance, change detection, GABA-mediated inhibition, corticofugal modulation, mismatch negativity

## Abstract

Deviancy detection in the continuous flow of sensory information into the central nervous system is of vital importance for animals. The task requires neuronal mechanisms that allow for an efficient representation of the environment by removing statistically redundant signals. Recently, the neuronal principles of auditory deviance detection have been approached by studying the phenomenon of stimulus-specific adaptation (SSA). SSA is a reduction in the responsiveness of a neuron to a common or repetitive sound while the neuron remains highly sensitive to rare sounds (Ulanovsky et al., 2003). This phenomenon could enhance the saliency of unexpected, deviant stimuli against a background of repetitive signals. SSA shares many similarities with the evoked potential known as the “mismatch negativity,” (MMN) and it has been linked to cognitive process such as auditory memory and scene analysis (Winkler et al., 2009) as well as to behavioral habituation (Netser et al., 2011). Neurons exhibiting SSA can be found at several levels of the auditory pathway, from the inferior colliculus (IC) up to the auditory cortex (AC). In this review, we offer an account of the state-of-the art of SSA studies in the IC with the aim of contributing to the growing interest in the single-neuron electrophysiology of auditory deviance detection. The dependence of neuronal SSA on various stimulus features, e.g., probability of the deviant stimulus and repetition rate, and the roles of the AC and inhibition in shaping SSA at the level of the IC are addressed.

## The auditory system is highly sensitive to deviant stimuli

An animal's behavioral responses to unexpected changes in the continuous flow of sensory, including acoustic, information are critical for its survival. These responses depend on the ability to detect deviancy in an ongoing stimulus. In order for the nervous system to determine whether a sound is deviant, there must be an ongoing storage of information about the sounds that have already occurred, as well as a comparison of new sounds with previous ones.

**Figure 1:**
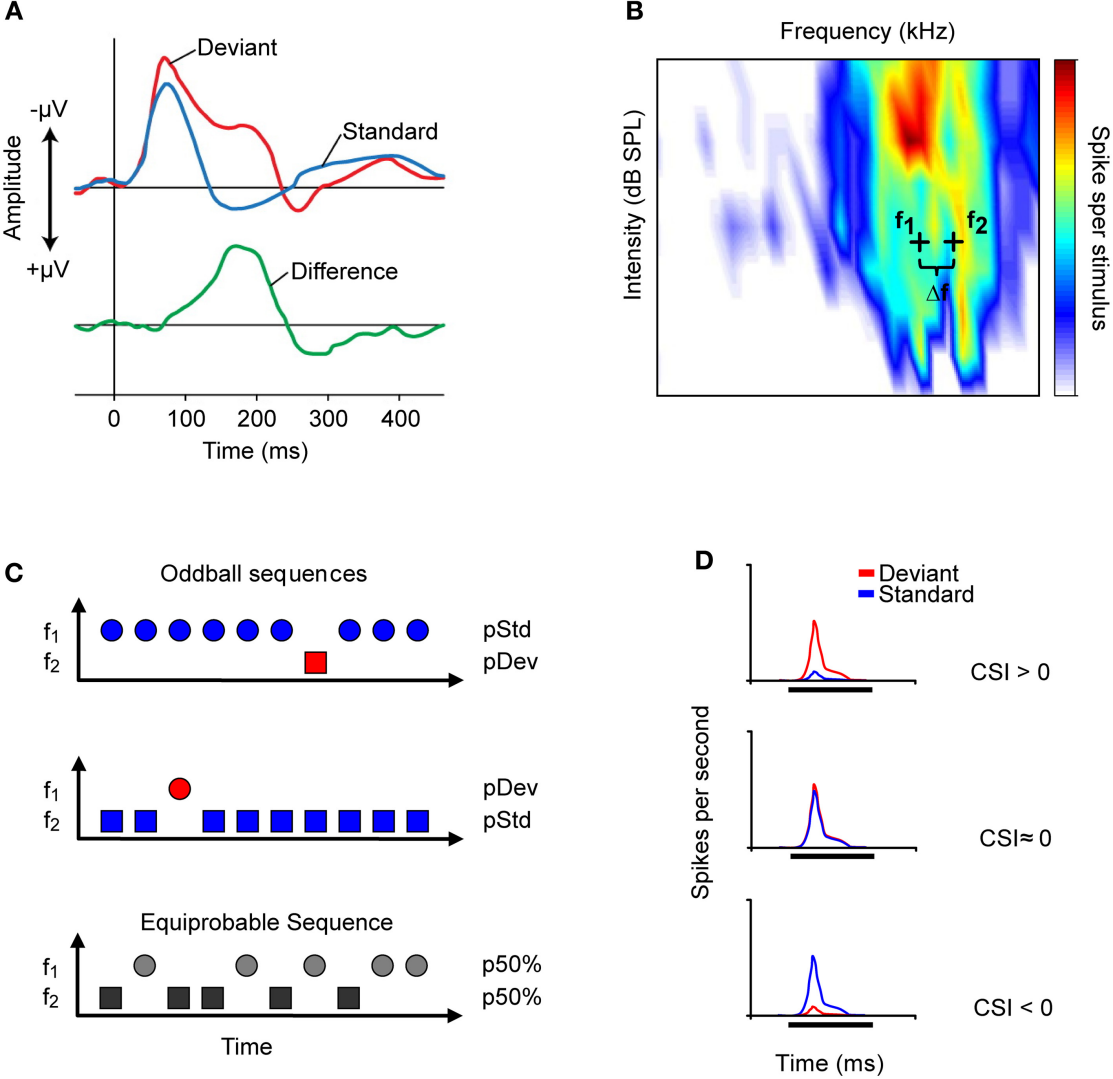
**Auditory change detection is studied by recording the evoked potentials of populations of neurons or the responses of single-units. (A)** Schematic representation of the evoked potential known as the mismatch negativity (MMN) recorded through an electroencephalogram. The MMN is associated with the detection of deviant auditory events. It is measured as the difference (green line) between the potential elicited by a sequentially repeated stimulus (standard, blue line), and that elicited by a sound “deviating” in any of its attributes (deviant, red line). The peak of the MMN wave usually occurs at 150–200 ms from the onset of the deviant stimulus. **(B)** Frequency response area (FRA, combination of frequencies and intensities capable of evoking a response) of an IC neuron. Based on the FRA, the experimenter chooses a pair of frequencies (*f*_1_ and *f*_2_, black crosses) with a fixed physical separation (Δ*f*) and at the same level to present in the oddball paradigm. Usually, in studies of the IC, a Δ*f* of 0.058, 0.144, or 0.53 octaves is used. **(C)** Representation of the oddball paradigm used to study the detection of frequency deviance. In one oddball sequence (top), one frequency (*f*_1_) is presented as the standard stimulus with a high probability of occurrence (blue symbol), while the second (*f*_2_) is the rarely occurring deviant stimulus (red symbol), interspersed randomly among the standards. In a second oddball sequence (middle), the relative probabilities of the two stimuli are reversed, with *f*_2_ as the standard (blue) and *f*_1_ as the deviant (red). As a control (bottom), both frequencies are presented with the same probability of occurrence [equiprobable condition; *p* (*f*_1_) = *p* (*f*_2_) = 50%]. **(D)** The common-SSA index (CSI) is a widely used metric to quantify the extent of SSA in the neuronal responses. The three PSTHs represents the response to deviant (red) and to standard (blue) tone of neurons exhibiting different values of CSI. The CSI compares the responses to deviant and to standard stimuli from the two oddball sequences by normalizing the responses in terms of spikes per stimulus (see text). The normalization corrects for the different number of presentations of the standard and deviant stimuli in each sequence. The possible values of the CSI range from +1 to −1, with positive values indicating that the response magnitude to the deviant is higher than that to the standard stimulus (upper PSTH); zero indicating that the two stimuli elicit equal responses (middle PSTH); and negative values indicating that the response to the standard stimulus is greater than that to the deviant stimulus (bottom PSTH).

Auditory deviance detection has been widely explored in human electroencephalogram (EEG) studies (for a review see Grimm and Escera, 2012). Those studies have shown that the waveform elicited by a deviant (low probability) stimulus differs from that elicited by a predictable (high probability) stimulus. Deviance detection has been associated with a particular evoked potential derived from the human EEG, namely the “mismatch negativity” (MMN; Näätänen et al., 1978; for recent review, see Näätänen et al., 2007). The MMN is measured as the difference between the evoked potential elicited to a sequentially repeated (high probability) stimulus, referred to as the “standard,” and that elicited by a rarely occurring (low probability) sound referred to as the “deviant” that differs in any of its attributes such as location, pitch, intensity, or duration (Figure 1A). The MMN usually peaks at 150–200 ms from the onset of a change. It has a frontocentral scalp distribution, with positive voltages at electrode positions below the Sylvian fissure, indicating generator sources located bilaterally on the supratemporal plane of the auditory cortex (AC) (Huotilainen et al., 1998) with an additional prefrontal contribution (Shalgi and Deouell, 2007). The MMN is useful for the study of deviance detection in humans because (1) it provides a reliable signal of auditory change detection (e.g., Escera et al., 2000), and (2) it can be recorded passively, i.e., with no specific instruction given to the subjects, which makes it suitable for studying non-cooperative populations, such as patients with cerebral lesions, neurodegenerative diseases, or psychiatric disorders (e.g., Duncan et al., 2009; Luck et al., 2011), as well as newborns (Carral et al., 2005a) and fetuses (Draganova et al., 2005).

Because deviance detection requires information storage and comparison over time, it could be thought of as a form of cognitive processing, or “primitive intelligence” (Näätänen et al., 2001). For this reason, it has been commonly assumed that deviance detection must be accomplished at the level of the cortex. This assumption has persisted not only for theoretical reasons, but also because it is difficult to pinpoint the site at which EEG waveforms are generated, especially in the case of subcortical structures. However, the fact that the MMN persists during sleep or anesthesia suggests that it is “preattentive” in origin (Tiitinen et al., 1994) and therefore could originate subcortically. This idea was largely untested and controversial until recently, as deviant occurrence is also reflected in the middle latency response (MLR) range of the evoked-activity, indicating that auditory change detection already occurs in early stages of human auditory processing (Slabu et al., 2010, 2012; Grimm et al., 2011). The MLR is a sequence of waveforms in the range of 12–50 ms from sound onset that precedes the well-studied MMN and it is generated by activation of subcortical areas as well as primary and secondary cortical areas (Grimm and Escera, 2012). Moreover, Slabu et al. (2012) showed that the human auditory brainstem is able to encode regularities in the recent auditory past that could be used for comparison to deviant events, and confirmed multiple anatomical and temporal scales of human deviance detection.

Finally, it is worth mentioning that MMN-like auditory-evoked potentials have also been recorded in laboratory animals in the AC (Javitt et al., 1994; Ruusuvirta et al., 1998; Eriksson and Villa, 2005; Astikainen et al., 2006, 2011; Tikhonravov et al., 2008; Nakamura et al., 2011) and subcortical structures: thalamus of the guinea pig (Kraus et al., 1994; King et al., 1995) and inferior colliculus (IC) of the rat (Patel et al., 2012). Overall, these studies suggest that deviance detection may be a basic property of the functional organization of the auditory system occurring on multiples levels along the auditory pathway (Grimm et al., 2011; Grimm and Escera, 2012). Nevertheless, identification of the neuronal microcircuits and functional mechanisms underlying deviance detection remains a challenge in auditory neuroscience.

## Stimulus-specific adaptation: a neural mechanism for detection of deviant stimuli

A decade ago, Nelken and colleagues (Ulanovsky et al., 2003) published a pioneering study that described a phenomenon similar to MMN and occurring at the cellular level in the mammalian AC. The single-neuron phenomenon was referred to as stimulus-specific adaptation (SSA) [a term originally coined by Movshon and Lennie (1979)] and it is proposed to be a neuronal mechanism that could be contributing to auditory deviance detection (Ulanovsky et al., 2003; Jääskeläinen et al., 2007). SSA is the reduction in the responsiveness of a neuron to a common or repetitive sound while the response decrement does not generalize to others sounds that are rarely presented. In this sense, SSA is a phenomenon of neuronal adaptation to the history of the stimulus rather than to the activity of the neuron (Nelken and Ulanovsky, 2007; Netser et al., 2011; Gutfreund, 2012). Although, MMN and SSA share several features such as their dependence on stimulation rate, they also greatly differ in their latencies and level of the neural structures involved (Nelken and Ulanovsky, 2007; Grimm and Escera, 2012). In addition, MMN is elicited by deviant stimuli immersed in complex forms of regularities (Carral et al., 2005b; Cornella et al., 2012; Recasens et al., 2012) that remained to be tested in single-unit recordings.

SSA is a widespread phenomenon in the brain exhibited by neurons of the visual (Woods and Frost, 1977; Sobotka and Ringo, 1994; Müller et al., 1999), somatosensory (Katz et al., 2006) and auditory (Ulanovsky et al., 2003; Malmierca et al., 2009a; Reches and Gutfreund, 2008; Anderson et al., 2009; Antunes et al., 2010) systems.

Auditory neurons that exhibit SSA for frequency deviance have been described in the IC (Pérez-González et al., 2005; Reches and Gutfreund, 2008; Malmierca et al., 2009a; Lumani and Zhang, 2010; Netser et al., 2011; Zhao et al., 2011), medial geniculate body (MGB) (Anderson et al., 2009; Yu et al., 2009; Antunes et al., 2010; Baüerle et al., 2011), and primary AC (Ulanovsky et al., 2003, 2004; Von Der Behrens et al., 2009; Taaseh et al., 2011). In the brainstem, SSA has been recently explored in the ventral and dorsal cochlear nucleus of the rat and the results suggest that all cochlear nucleus neurons tested lack SSA (Ayala et al., 2012). Thus, the IC is the earliest center where SSA has been described (Malmierca et al., 2009a), although it remains to be tested whether or not other brainstem nuclei located in between the cochlear nucleus and IC show some degree of SSA. In this review, we will focus on a description of SSA occurring at the IC, which is the major auditory station in the midbrain for the integration of ascending, descending, intrinsic, and commissural inputs (Malmierca, 2003; Malmierca et al., 2003, 2005a,b, 2009b; Cant and Benson, 2006; Loftus et al., 2010; Malmierca and Hackett, 2010; Malmierca and Ryugo, 2011).

## Stimulus-specific adaptation in single-neuron response

Most of the studies of SSA in the IC have been carried out in the anesthetized rat (Pérez-González et al., 2005, 2012; Malmierca et al., 2009a; Lumani and Zhang, 2010; Zhao et al., 2011; Patel et al., 2012). Initially, Pérez-González et al. (2005) found that cortical IC neurons of the rat show adaptation to the repetitive stimulation of an acoustic parameter, but they resume firing if the sound parameter was changed. Responses similar to those reported by Pérez-González and colleagues were originally described in the midbrain of frogs (Bibikov, 1977; Bibikov and Soroka, 1979).

More recent studies have explored sensitivity to frequency deviance using an “oddball paradigm,” similar to that used to record MMN responses in human studies (Näätänen, 1992). In this paradigm two tones are selected within the frequency response area of the neuron (Figure 1B) and randomly presented with different probabilities of occurrence. The high probability tone is usually referred to as the standard stimulus; interspersed among the standard stimuli are the low probability or deviant stimuli. In a second sequence of stimulation, the relative probabilities are reversed so that both frequencies are presented as standard and deviant. Usually, the neuronal responses are also recorded under an equiprobable condition, in which both frequencies have the same probability of occurrence (Figure 1C).

The amount of SSA is quantified by an index that reflects the extent to which a neuron responds to tones when they are presented as the deviant stimulus compared to when they are presented as the standard stimulus. This index is referred as the common SSA index (CSI) defined as CSI = [*d*(*f*_1_) + *d*(*f*_2_) − *s*(*f*_1_) − *s*(*f*_2_)]/[*d*(*f*_1_) + *d*(*f*_2_) + *s*(*f*_1_) + *s*(*f*_2_)], where *d(f)* and *s(f)* are responses measured as spike rate to frequencies *f*_1_ or *f*_2_ used as either the deviant (*d*) or standard (*s*) stimulus. The CSI values range from −1 to +1, being positive when the response to the deviant stimulus is stronger, and negative when the standard stimulus evokes more spikes (Figure 1D).

**Figure 2:**
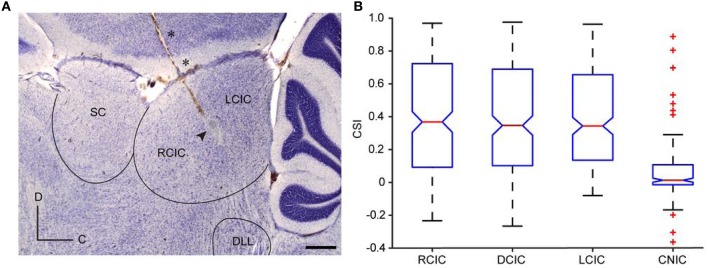
**Anatomical location of SSA in the IC. (A)** Photomicrography showing a sagittal section of the IC with a typical electrode track (asterisks) and the electrolytic lesion generated (arrowhead). Scale bar = 500 μm. C, caudal; D, dorsal. **(B)** Box plot with the median value (red line) of CSI sorted by anatomic regions. The blue box delimits the 25^th^ and 75^th^ percentile and dashed lines show the most extreme data points not considered outliers. Red crosses indicate outliers. Cortical regions (RCIC, DCIC, and LCIC) are significantly different from the CNIC (Kruskal–Wallis test, *p* < 0.001). Reproduced from Duque et al. (2012).

SSA to frequency deviance has been found to be stronger in the non-lemninscal regions, i.e., in the dorsal (DCIC), rostral (RCIC), and lateral cortices (LCIC), than in the central nucleus (CNIC) (Pérez-González et al., 2005; Malmierca et al., 2009a, 2011; Lumani and Zhang, 2010; Duque et al., 2012) (Figure 2). Studies of specific adaptation have also been reported in the awake barn owl (Reches and Gutfreund, 2008; Netser et al., 2011) and bat (Thomas et al., 2012).

Sensitivity to intensity and duration deviance has been observed in the AC (Ulanovsky et al., 2003; Farley et al., 2010) but it is not as robust as frequency deviance. These other stimulus features, i.e., intensity and duration, have not been tested under the oddball paradigm in the IC, but it seems likely that subcortical neurons that show SSA to frequency may also be able to detect deviance in other stimulus dimensions, as occurs with neurons of the midbrain of avians. Neurons in the optic tectum (analogous to the superior colliculus of mammals) of the barn owl exhibit SSA to sound frequencies, amplitude, and interaural time and level difference (Reches and Gutfreund, 2008).

The great majority of neurons with high levels of SSA display transient onset responses and have low or absent spontaneous activity in anesthetized rats (Pérez-González et al., 2005, 2012; Malmierca et al., 2009a; Lumani and Zhang, 2010; Duque et al., 2012). This finding is consistent with a higher incidence of SSA in the non-lemniscal IC since a large proportion of neurons in the dorsal regions of the IC have onset responses (Reetz and Ehret, 1999; LeBeau et al., 1996). Moreover, for adapting neurons with other types of responses, i.e., on-sustained and on-off (Rees et al., 1997), the largest difference between responses to deviant and standard stimuli is signaled by the onset component (Malmierca et al., 2009a; Duque et al., 2012).

**Figure 3:**
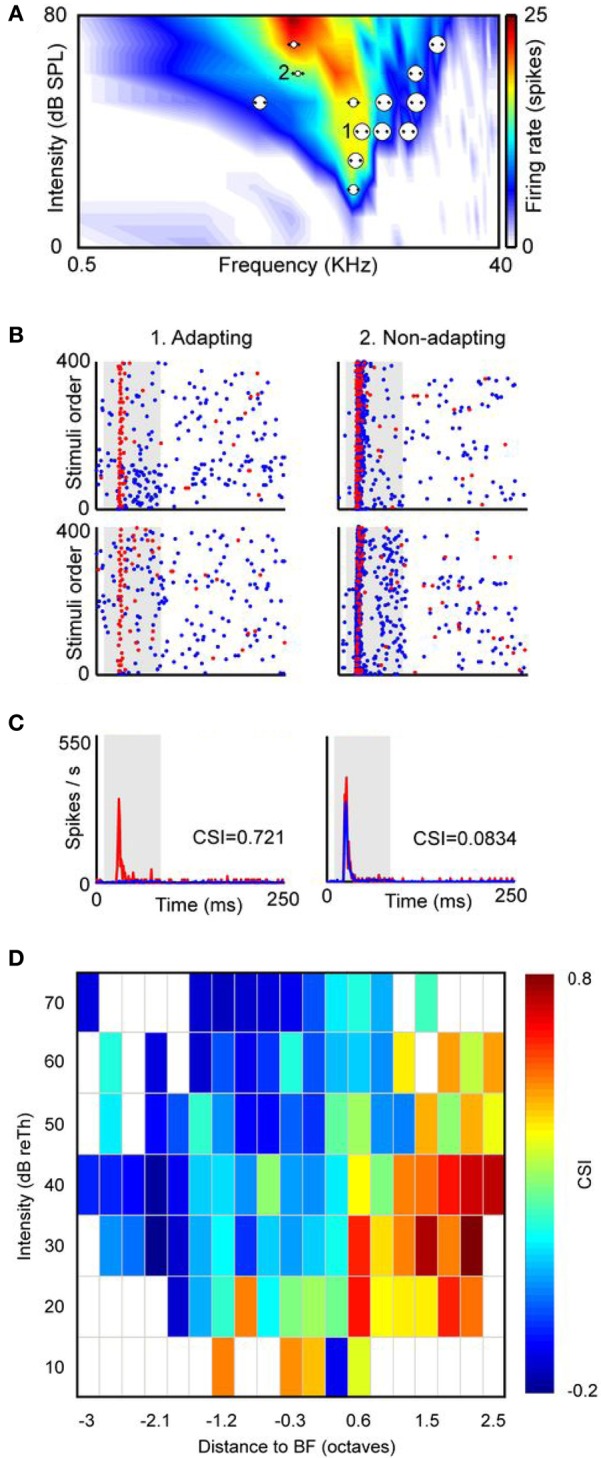
**SSA is not homogeneously distributed within the frequency response area of IC neurons. (A)** Example of a neuron with a broad V-shaped response area and the distribution of the several pairs of frequencies presented under the oddball paradigm (dots). Each pair of dots is associated to a circle the size of which is proportional to the level of CSI evoked. An example of an adapting pair of frequencies (i.e., frequencies that elicited SSA) is marked as 1 and another example of a non-specifically adapting pair of frequencies is marked as 2. **(B)** Dot raster plots obtained with the adapting pair (left panels) and with the non-specifically adapting (right panels) pair of frequencies. The blue dots represent spikes evoked by the standard stimulus (90% probability), while the red dots represent those evoked by the deviant stimulus (10% probability). Stimulus presentations are accumulated in the temporal domain along the vertical axis. In the adapting examples red dots are more visible because of the specific decrease of the response to the standard stimulus. The upper panels are the ones obtained when *f*_1_ was the standard tone and *f*_2_ was the deviant, and the bottom panels represent the response when the relative probabilities were inversed, that is, *f*_1_ was the deviant and *f*_2_ the standard. **(C)** Averaged PSTH for both frequencies when deviant (red) or standard (blue). CSI values obtained in each pair of frequencies are showed as insets in the PSTH. The shaded backgrounds in the dot raster and PSTH plots indicate the duration of the stimulus. **(D)** Distribution of the CSI values sorted relatively to the best frequency (BF) and the threshold (Th) of the response area of a sample of IC neurons (almost 80% are from the cortical region, *n* = 124). Higher CSI values are confined to the high-frequency edge and at low sound intensities. Reproduced from Duque et al. (2012).

Another feature of neurons that exhibit SSA is their broad frequency response area (Malmierca et al., 2009a; Duque et al., 2012). In the IC of the rat, neurons in the DCIC and RCIC regions possess widespread dendritic arbors (Malmierca et al., 1993, 1995, 2011), and broader frequency tuning than the CNIC (Syka et al., 2000; Duque et al., 2012). A possible functional consequence of neurons with large dendritic arbors is the integration of inputs over a broad frequency range. Among cortical IC neurons the broader the frequency response area the higher the level of SSA observed (Duque et al., 2012). In the bat IC, SSA is present in a subset of non-specialized neurons which are broadly tuned to frequency and non-selective for spectrotemporal pattern (Thomas et al., 2012) suggesting a complex input processing. Furthermore, SSA is not a property homogeneously distributed throughout the neuron's frequency response area. Duque et al. (2012) compared the degree of SSA at multiple combinations of frequencies and intensities in single-unit recordings in the IC of the anesthetized rat. They found that adapting neurons exhibit stronger SSA at the high frequency edge of the response area and low sound intensities (Figure 3). This study concluded that SSA is not constant within the neuronal receptive field, and therefore is not a characteristic property of the neuron, instead the neuron's inputs contribute to its generation.

In addition to encoding deviance by spike count, IC neurons can also encode deviance information through their spike timing. In neurons that exhibit SSA, the first spike latency (FSL) in the response evoked by the deviant tone is shorter than that evoked by the standard tone (Malmierca et al., 2009a; Zhao et al., 2011; Duque et al., 2012). This phenomenon is known as “latency adaptation” and seems to be a unique feature of subcortical neurons (Malmierca et al., 2009a; Antunes et al., 2010; Duque et al., 2012). The neurons in the DCIC that show SSA have much longer FSLs than neurons in the CNIC (Lumani and Zhang, 2010). Thus, temporal coding appears to play a key role in the signaling of deviance.

## Stimulus-specific adaptation and its relationship to stimulation parameters

A hallmark of SSA in the different auditory areas is its sensitivity to a variety of stimulus parameters such as the deviant probability, the frequency separation, and the time interval (stimulation rate) between stimuli (Ulanovsky et al., 2003; Malmierca et al., 2009a; Yu et al., 2009; Von Der Behrens et al., 2009; Antunes et al., 2010; Zhao et al., 2011). This dependency is also present for the processing of other stimulus features such as interaural time- and level-differences and amplitude deviants (Ulanovsky et al., 2003; Reches and Gutfreund, 2008).

The manipulation of the probability of occurrence of the deviant and, consequently, of the standard stimulus has a strong effect on the extent of SSA observed (Malmierca et al., 2009a; Patel et al., 2012). Deviant probabilities of 30 and 10% have been explored; SSA increases as the deviant probability decreases. Thus, neurons are sensitive to stimulus probability with greater sensitivity to tones that are less likely to occur (Malmierca et al., 2009a).

The sensitivity to deviance increases proportionally with the extent of physical separation between tones. The frequency contrast is expressed as Δ*f* = (*f*_2_ − *f*_1_)/(*f*_2_ × *f*_1_)^1/2^; where *f*_2_ and *f*_1_ correspond to the frequencies tested (Ulanovsky et al., 2003) (Figure 1B). Most studies of SSA have employed pure tones centered on the best (Malmierca et al., 2009a; Pérez-González et al., 2012) or characteristic frequency (Zhao et al., 2011) of the neurons. The differential adaptation of the responses to deviant and standard stimuli is more prominent when Δ*f* increases from 0.04 to 0.1 to 0.37 (0.058, 0.144, and 0.531 octaves, respectively) (Malmierca et al., 2009a). Neurons with strong SSA in the IC, as in the MGB and primary AC, show hyperacuity, that is, a strong and highly sensitive adaptation for frequency ratios as small as 4% (Δ*f* = 0.04). This frequency ratio is smaller than the width of their frequency response areas (Ulanovsky et al., 2004; Moshitch et al., 2006; Malmierca et al., 2009a).

**Figure 4:**
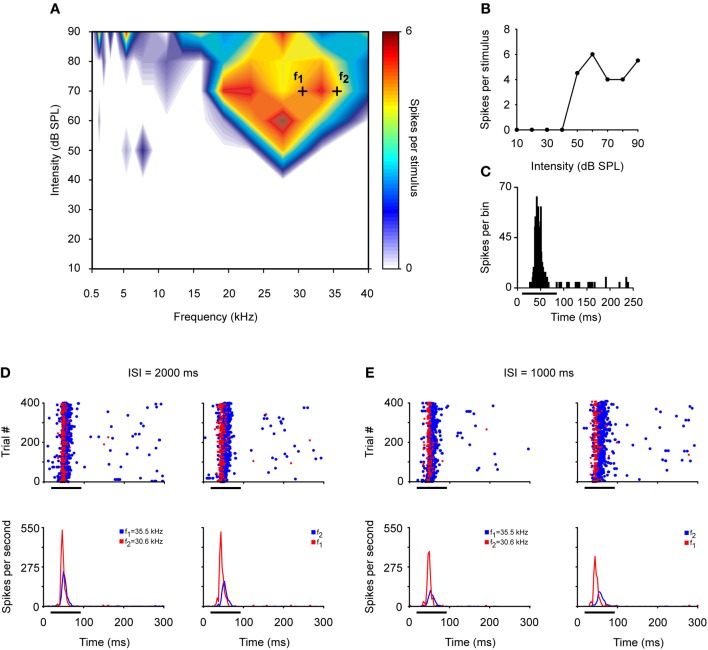
**Example of an IC neuron that exhibits SSA at low repetition frequencies. (A)** Broad frequency response area displayed in color code according to the strength of the response. Black crosses represent a frequency pair—*f*_1_ (30.6 kHz) and *f*_2_ (35.5 kHz)—with a physical separation of 0.216 octaves (Δ*f* = 0.15) at 70 dB SPL. **(B)** The neuron exhibits a non-monotonic rate-level function at its best frequency (27.1 kHz). **(C)** PSTH of the accumulated responses to all the frequencies (0.5–40 kHz) and intensities (10–90 dB SPL) presented (1 ms bins). **(D)** Top panels. Dot rasters of the response to oddball sequences of 400 stimulus presentations with a repetition rate of 0.5 Hz (ISI = 2000 ms). The frequencies used as the deviant stimulus (probability of 10%, red dots) and the standard or repetitive stimulus (probability of 90%, blue dots) are reversed in the left and right panels. The bottom panels show the corresponding normalized PSTHs to deviant (red line) and standard (blue line) stimuli. **(E)** Response to the oddball sequences presented at a higher repetition rate of 1Hz (ISI = 1000 ms). Format same as for panel **(D)**.

**Figure 5:**
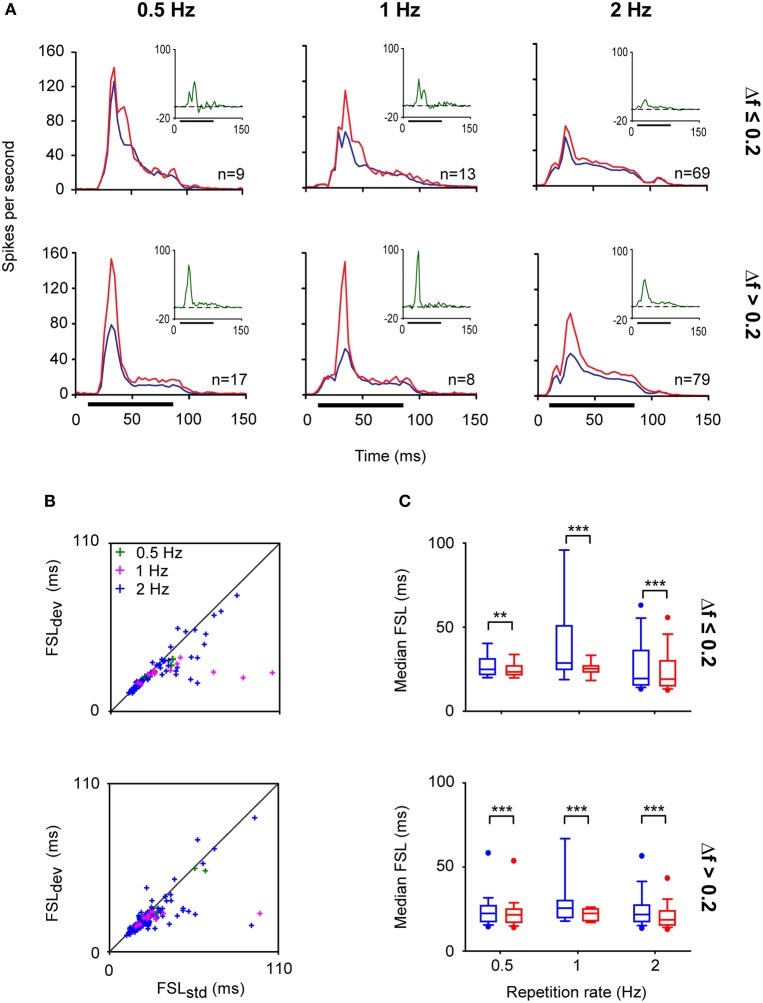
**IC neurons exhibit SSA even at very low repetition rates. (A)** Population PSTHs of the IC neuronal responses to a deviant (red line) and to a standard (blue line) stimulus presented at different repetition rates; 0.5 (ISI = 2000 ms), 1 (ISI = 1000 ms), and 2 Hz (ISI = 500 ms). The physical separations between frequencies were grouped into Δ*f* ≤ 0.2 (≤ 0.288 octaves) (top panels) and Δ*f* > 0.2 (bottom panels). The firing rates of individual neurons were averaged and normalized to account for the different number of stimulus presentations due to the different probabilities of the tones (deviant; 10%, standard; 90%). The insets shown in the right upper quadrants of each graph correspond to the averaged difference between the responses to the deviant and standard stimuli (green lines). The horizontal black bars indicate the duration of the stimulus. **(B)** Scatter plots of the first spike latency (FSL) of the neuronal responses evoked by *f*_1_ and *f*_2_ when they were the deviant (FSL_dev_) or the standard (FSL_std_) stimulus according to their frequency separation (Δ*f* ≤ 0.2 and and Δf > 0.2) and across different repetition rates (0.5, 1, and 2 Hz). **(C)** Box plots of the population FSL indicating that even at very low repetition rates the FSLs evoked by the deviant tone (red boxes) are shorter than those evoked by the standard stimulus (blue boxes). (Signed-Rank Test; ^**^p < 0.01, ^***^p < 0.001).

The highest levels of SSA are elicited by IC neurons at an ISI of 250 ms with a deviant probability of 10% compared to shorter (125 ms) or longer ISIs (500 ms) (Malmierca et al., 2009a). Few studies have explored SSA at very long ISIs. It has been reported that IC neurons are still capable of detecting frequency deviants at ISIs up to 1 s (repetition rate of 1 Hz) (Pérez-González et al., 2005; Reches and Gutfreund, 2008; Zhao et al., 2011). Indeed, we have recorded neurons that exhibit SSA at even slower repetition rates (ISI = 2000 ms) (Malmierca et al., 2010). Figure 4 illustrates an example of the response of an IC neuron that exhibits SSA in the anesthetized rat. This neuron was very broadly tuned with a complex non-monotonic rate-level function and a transient onset firing pattern (Figures 4A–C). For this neuron, a pair of frequencies (black crosses; *f*_1_ and *f*_2_) with a physical separation of 0.216 octaves (Δ*f* = 0.15) was chosen for presentation in the oddball paradigm with an ISI of either 2000 or 1000 ms. Figures 4D,E depict the responses to the deviant and standard stimuli as dot rasters and the corresponding peri-stimulus time histogram (PSTH) obtained with 0.5 and 1 Hz repetition frequencies, respectively. Under these conditions, the neuron showed SSA (CSI: 0.3–0.4), resulting in higher-evoked spiking to the deviant frequency (red color). Figure 5A shows the average PSTHs of a subset of neurons recorded with ISIs of 2000, 1000, and 500 ms. It is evident that there is an increasing difference between responses to deviant and standard tones as the frequency separation is increased (insets). Under these extreme stimulation rates, the FSLs evoked by the deviant tone were earlier than those evoked by the same tone when it was used as the standard, suggesting that the cellular mechanisms that discriminate between deviant and standard responses are functional at the temporal scale of seconds (Figures 5B,C). As highlighted by Nelken and Ulanovsky (2007), the neuronal mechanisms for deviance detection become more important at more extreme stimulation values, e.g., slow stimulation rates and very similar stimulus frequencies.

Long interstimulus intervals affect the long-term dynamics of SSA, prolonging the time course of adaptation from several seconds at ISI of 125 ms, to tens of seconds when ISI is increased to 1000 ms. The frequency resolution of neurons is also modified by the repetition rate (Malmierca et al., 2009a; Antunes et al., 2010; Zhao et al., 2011). Frequencies separated by Δ*f* = 0.04 do not elicit SSA in the IC or in the MGB of the rat (Antunes et al., 2010) when tested with the longest ISI of 2000 ms. Together these data indicate that the timing between stimuli affect both the extent and frequency resolution of SSA.

SSA at the time scale of 2000 ms has been found in primary AC, but cortical neurons do not adapt at ISIs longer than 2000 ms (Ulanovsky et al., 2003). SSA also occurs at an ISI of 2000 ms in the MGB, a mandatory processing station between the IC and the AC (Antunes et al., 2010). The presence of SSA in subcortical structures such as the IC and MGB on a time scale of the magnitude of seconds, similar to the time scale of cognition (Ulanovsky et al., 2003, 2004; Nelken and Ulanovsky, 2007), suggests that deviance detection at these early neuronal stages could be contributing to the perceptual organization of the components of complex auditory stimuli (Winkler et al., 2009) and to the change detection recorded in local field potentials (Slabu et al., 2010; Grimm et al., 2011). This temporal scale is consistent with the duration of sensory (echoic) memory in monkeys and humans, which is estimated to be in the order of a few seconds (Javitt et al., 1994; Näätänen and Escera, 2000).

**Figure 6:**
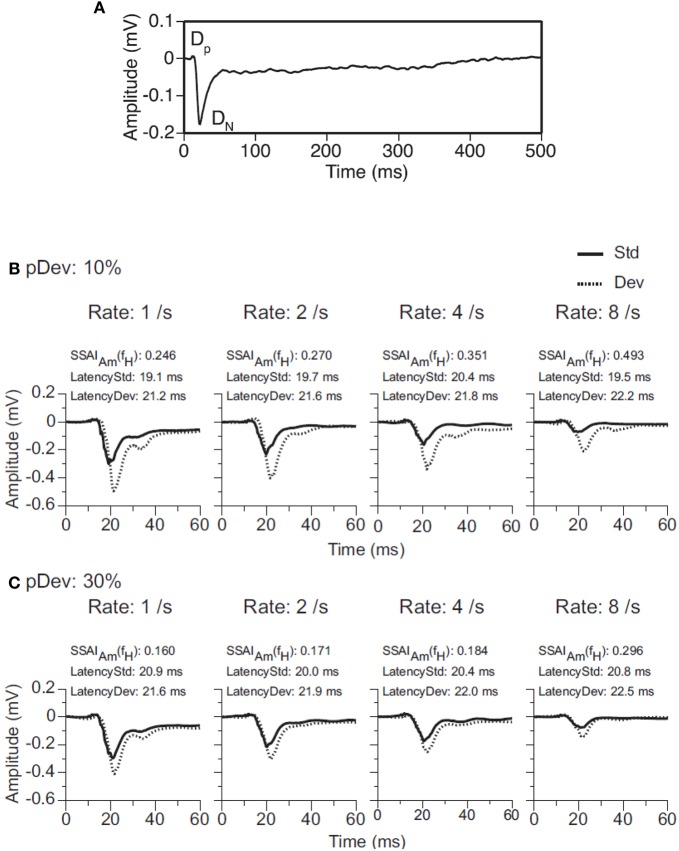
**Evoked local field potentials (LFP) recorded in the DCIC. (A)** An average LFP is a waveform with a relatively small positive deflection (*D*_P_) followed by a large negative deflection (*D*_N_). Together, the two deflections are about 40 ms in total duration. (**B,C**) Evoked LFPs in response to a tone burst presented as standard stimulus (Std, solid line) or deviant stimulus (Dev, dotted line) in a pair of oddball sequences with probabilities of occurrence of the deviant of 10% (panel **B**) or 30% (panel **C)**. Data were obtained for pairs of tones (*f*_1_ = 2.25 kHz and *f*_2_ = 3.24 kHz.) separated by a Δ*f* = 0.37 and presented at 1, 2, 4 and 8 Hz. The BF of the recording site was 2.7 kHz. An amplitude-based, frequency-specific, stimulus-specific adaptation index [SSAI_Am_(*f*_X_)], along with the peak latencies of the responses evoked by the sound as standard and deviant stimuli, is shown at the top of each graph. Reproduced from Patel et al. (2012).

The same systematic dependence on deviant probability, frequency contrast, and repetition rate seen in single neurons is also present in the activity of ensembles of neurons in the IC (Figure 6). In a recent study the evoked local field potentials of DCIC neurons of the rat were recorded while stimulating with tone bursts under an oddball paradigm (Patel et al., 2012). The auditory response is a waveform with a relatively small positive deflection (*D*_P_) followed by a large negative reflection (*D*_N_) (Figure 6A). The degree of SSA is quantified by comparing the amplitude of the waves evoked by the deviant and standard stimuli using an amplitude-based, frequency-specific, SSA index for *D*_P_ and *D*_N_ (SSAI_*Am*_). As expected, the amplitude of the local field potential is larger to a tone burst presented as deviant than as standard, and the difference is greater when the deviant stimulus is less likely to occur (deviant probability of 10% rather than 30%) (Figures 6B,C). The difference between responses to deviant and standard tones occurs after the initial rising phase of the dominant deflection (*D*_N_), and no difference is elicited at the beginning of the response in *D*_P_. Overall, the results by Patel et al. (2012) suggest that frequency deviance coding is detectable in ensembles or neuronal microcircuits of subcortical neurons as an evoked response with a larger amplitude and longer peak latency. There are some differences, however, compared to the SSA observed in single-units. The frequency separation required to elicit SSA is greater (Δ*f* = 0.37 rather than Δ*f* = 0.1); the strongest SSA is elicited with a higher repetition rate (8 Hz); and the peak latency of the response to standard tone bursts is shorter than that to the deviant tone burst (contrary to the shorter latencies for deviants in single-unit recordings). As the authors suggested, these differences may be attributed to the fact that an evoked local field potential reflects a weighted average of voltage changes generated by multiple excitatory and inhibitory events in the vicinity of the recording electrode so that individual differences among neurons are largely averaged out. The short latency of responses in subcortical nuclei suggests that these responses could contribute to the earliest components of the evoked potential associated with the occurrence of a deviant acoustic event (Pa waveform in the MLR; peak at about 30 ms) (Grimm and Escera, 2012).

## Corticofugal modulation

It was originally proposed that auditory SSA has a cortical origin and is propagated to subcortical nuclei through direct corticofugal projections (Ulanovsky et al., 2003; Nelken and Ulanovsky, 2007). Indeed, in the IC and MGB, the strongest SSA has been described in the extralemniscal regions (cortical regions of the IC: Malone et al., 2002; Pérez-González et al., 2005; Malmierca et al., 2009a; Lumani and Zhang, 2010; medial division of the MGB: Antunes et al., 2010), which operate under strong cortical control (Loftus et al., 2008; He and Yu, 2010; Lee and Sherman, 2011; Malmierca and Ryugo, 2011).

In a recent study, Anderson and Malmierca (2013) addressed whether SSA in the IC is dependent upon the AC for its generation. The authors reversibly deactivated the AC by cooling through a cryoloop device (Lomber et al., 1999; Lomber and Malhotra, 2008) and recorded the changes in SSA sensitivity exhibited by IC neurons. This technique has been successfully used to study the influence of the corticofugal system in the auditory system, including a study of sensitivity in IC neurons to cues for spatial position (Nakamoto et al., 2008) and sensitivity to deviance in the MGB (Antunes and Malmierca, 2011).

**Figure 7:**
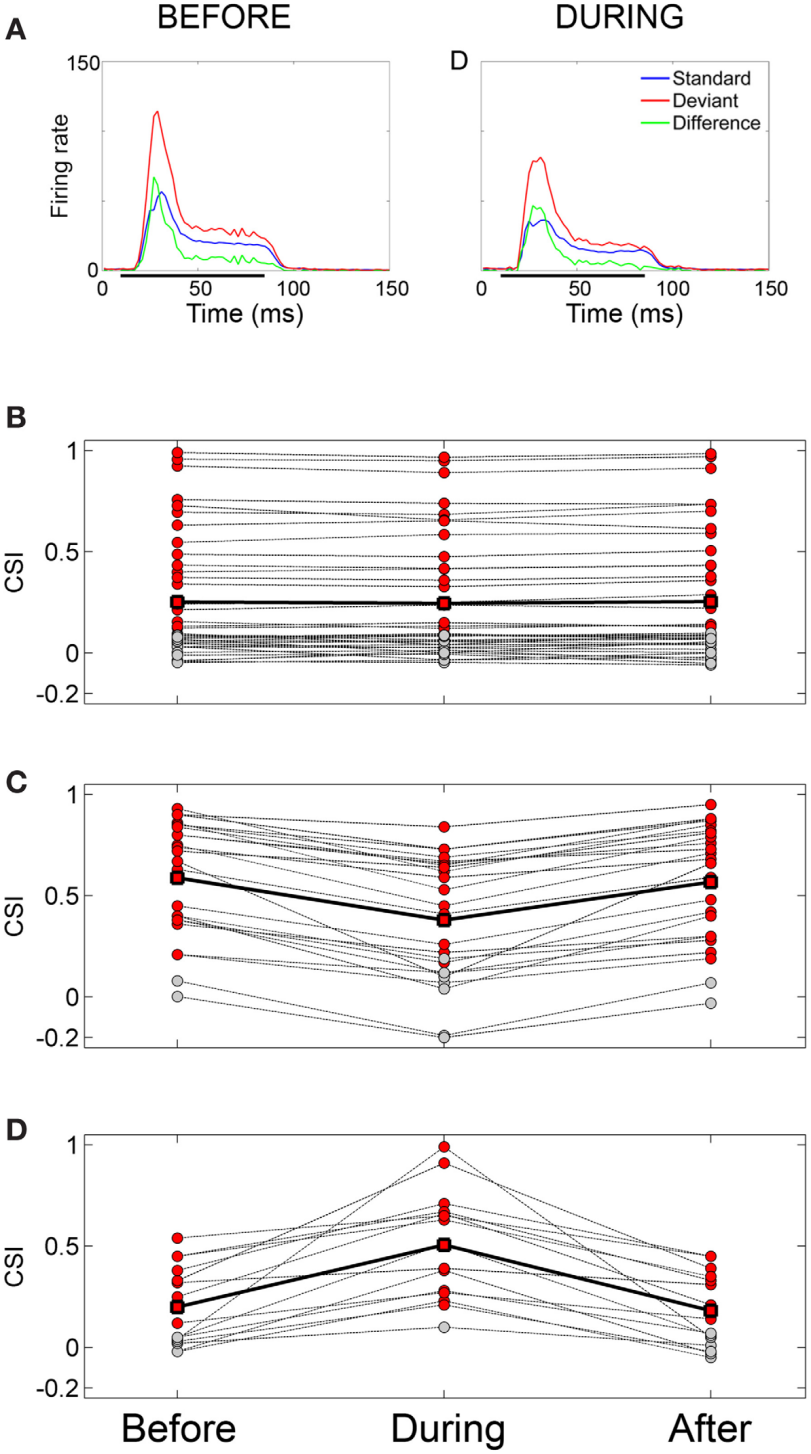
**Effect of cortical deactivation on SSA exhibited by IC neurons. (A)** Mean population PSTHs from IC recordings (*n* = 82) in response to tones presented as standard (blue) or deviant (red) before and during cooling the ipsilateral AC. The difference between the two is plotted in green. Deactivation of the auditory cortex does not eliminate the deviant saliciency in the averaged signal. **(B)** Population of neurons which showed no significant change in SSA during cooling, red circles indicate significant SSA, those in gray indicate non-significant CSI values. The solid line connected with square symbols indicates the mean CSI values for the three conditions. **(C)** Population which shows a decrease in SSA during cooling. Note that on cooling some neurons cease to show significant SSA. **(D)** Population which shows an increase in SSA during cooling. Note that on cooling some neurons which previously did not show significant SSA now become significant. Figure layout in **(C)** and **(D)** is the same as in **(A)**. Reproduced from Anderson and Malmierca (2013).

**Figure 8:**
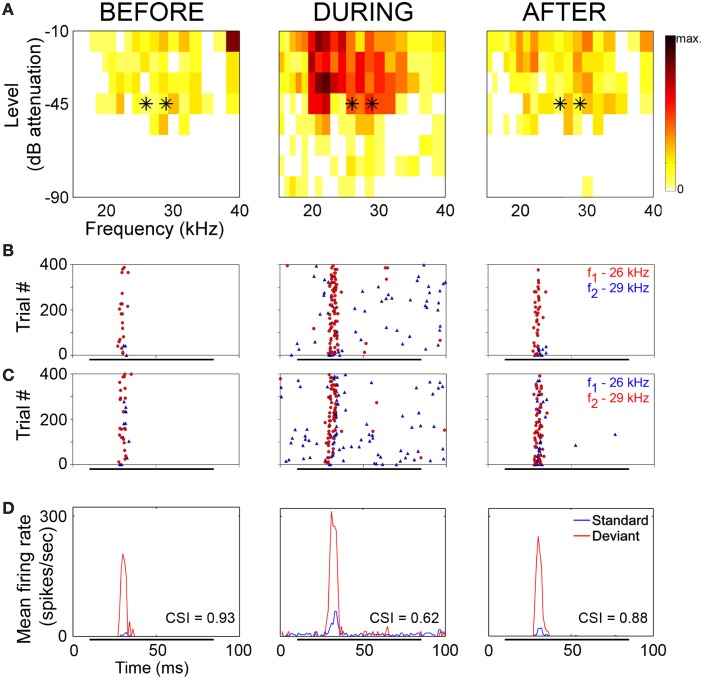
**Example of an IC neuron which shows a decrease in its adaptive properties with cooling. (A)** Frequency response areas recorded before (left), during (middle), and after (right) cooling. Black asterisks indicate the frequencies used in the oddball paradigm, firing rate (spikes/sec) indicated by the color scale which applies to all response areas in this figure. **(B)** Raster plots showing response to *f*_1_ (lower frequency) as deviant (red circles) and *f*_2_ (higher frequency) as standard (blue triangles). **(C)** Raster plots showing response to *f*_1_ as standard (blue triangles) and *f*_2_ as deviant (red circles). **(D)** PSTH showing mean response to standard (blue) and deviant (red) stimuli. CSI values for each period are superimposed onto the relevant PSTH. Reproduced from Anderson and Malmierca (2013).

**Figure 9:**
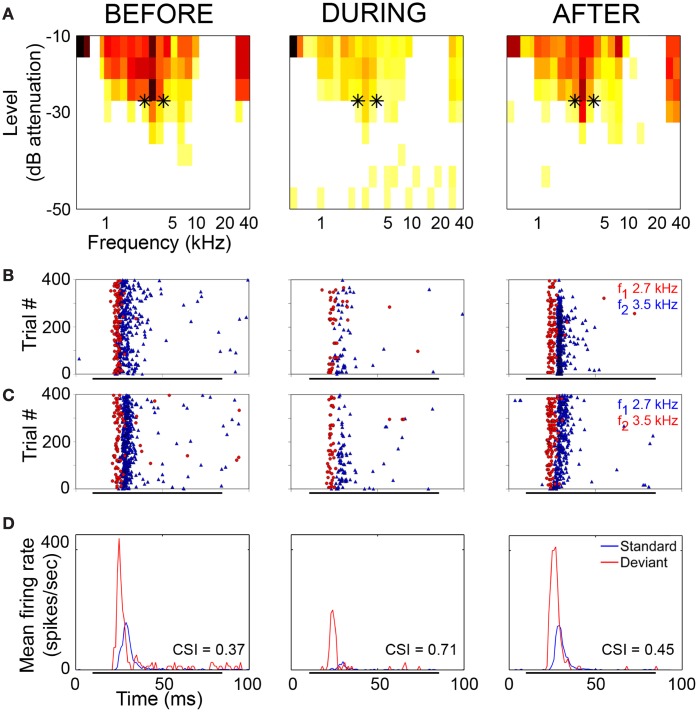
**Example of an IC neuron which shows stimulus-specific adaptation under normal conditions and increased SSA during the period of cortical cooling. (A)** Frequency response areas recorded before (left), during (middle) and after (right) cooling of AC. Black asterisks indicate the frequencies used in the oddball paradigm and the firing rate (spikes/sec) are indicated by the same colour scale that appears in Figure 8A. **(B)** Raster plots showing response to f_1_ (lower frequency) as deviant (red circles) and f_2_ (higher frequency) as standard (blue triangles). **(C)** Raster plots showing response to f_1_ as standard (blue triangles) and f_2_ as deviant (red circles). **(D)** PSTH showing mean response to standard (blue) and deviant (red) stimuli. CSI values for each period are superimposed onto the relevant PSTH. Reproduced from Anderson and Malmierca (2013).

The neuronal responses to the oddball paradigm (with a deviant probability of 10% and a frequency separation between deviant and standard stimuli of 0.531 octaves) were recorded before, during and after the cooling of the AC. At the population level, the main finding was that deactivation of the ipsilateral AC did not eliminate SSA exhibited by IC neurons. A decrease in firing rate to both the deviant and standard stimuli was observed during the cooling condition, but the response was still higher to the deviant stimulus (Figure 7A). Thus, the deviant salience in the IC was preserved even after the deactivation of cortical inputs. Interestingly, at the single-neuron level Anderson and Malmierca identified IC neurons that showed SSA and were insensitive to the cooling of the AC (Figure 7B). Those neurons exhibited different levels of SSA covering the full CSI spectrum, from zero to one. On the other hand, the adaptive properties of about half of the IC neurons with SSA (52%) were differentially affected throughout the period of cortical cooling, increasing (Figure 7C) or decreasing (Figure 7D) their SSA sensitivity. Examples of single neurons are shown in Figures 8 and 9. During the cooling period, the neuron displayed in Figure 8 increased its response area (Figure 8A), and its spontaneous and evoked firing rate. The increase was greater in response to the standard presentations (by a factor of seven) (Figures 8B–D, blue) than to the deviant ones (by a factor of three) (Figures 8B–D, red), resulting in a drop of its CSI (Figure 8D). On the other hand, the neuronal response illustrated in Figure 9 exhibited a disproportionately decrease in the firing rate with an almost extinguished response to the standard stimulus, thus, increasing its CSI. The authors suggest that the disproportionate changes in the neuron's firing rate to standard or deviant stimulus caused by the cortical inactivation may indicate the occurrence of a gain control modulation similar to that observed in the MGB under a similar manipulation (Antunes and Malmierca, 2011) and elicited by GABA_A_-mediated inhibition in the IC (Pérez-González and Malmierca, 2012; Pérez-González et al., 2012) which is clearly compatible with the “iceberg effect” notion described by, e.g., Isaacson and Scanziani, (2011, see below).

Overall, a decrease in SSA was the predominant response, although the majority of IC neurons continued to show significant SSA after the deactivation of the cortical inputs. The extreme effects of cortical cooling were exhibited by (1) IC neurons that lost their pre-existing deviance sensitivity during the cooled condition and (2) non-adapting neurons that began to exhibit SSA. The existence of sets of IC neurons differentially affected by the AC deactivation suggests that the ipsilateral AC may relay SSA to a small group of neurons, but that corticofugal inputs do not account significantly for the SSA exhibited by the majority of adapting neurons. Using the same cooling technique, Antunes and Malmierca (2011) demonstrated that SSA persisted in the MGB neurons regardless of the lack of functional corticofugal feedback, suggesting that SSA is inherited through lower input channels in a bottom-up manner and/or generated *de novo* at each level of the auditory pathway. Pharmacological manipulation in the IC suggests that local circuits may operate intrinsically to shape SSA at this neuronal station (Pérez-González and Malmierca, 2012; Pérez-González et al., 2012; see below).

## Role of inhibitory inputs in shaping SSA and possible mechanisms underlying SSA in the IC

It is well known that the IC integrates ascending and descending inputs from multiple sources (Malmierca, 2003; Malmierca and Ryugo, 2011) and possesses a dense and complex microcircuitry of local connections (Malmierca et al., 2003, 2005a, 2009b; Malmierca and Hackett, 2010). The IC is a major center for the convergence of both excitatory and inhibitory inputs and for combination of information across frequency-specific channels, especially in the non-lemnical regions (Malmierca et al., 2011). Inhibitory neurotransmission in the IC is mediated by GABAergic and glycinergic receptors (Palombi and Caspary, 1996; Caspary et al., 2002; Sivaramakrishnan et al., 2004; Ingham and McAlpine, 2005; Hernández et al., 2005; Malmierca et al., 2005a; Merchán et al., 2005). GABAergic inputs come from several sources, including bilateral projections from the dorsal nucleus of the lateral lemniscus and ipsilateral projections from the ventral nucleus of the lateral lemniscus as well as intrinsic and commissural GABAergic IC neurons (Hernández et al., 2005; Malmierca et al., 2003, 2005a). Glycinergic inputs originate from the ipsilateral lateral superior olive and from the ipsilateral ventral nucleus of the lateral lemiscus (Kelly and Li, 1997; Moore et al., 1998; Riquelme et al., 2001). Pharmacological manipulation of inhibitory neurotransmitters has strong effects on neuronal response area (LeBeau et al., 2001), firing rate (Palombi and Caspary, 1996), temporal response properties (LeBeau et al., 1996), tuning for sound duration (Casseday et al., 1994, 2000) as well as for frequency (Koch and Grothe, 1998) and amplitude modulation (Caspary et al., 2002).

**Figure 10:**
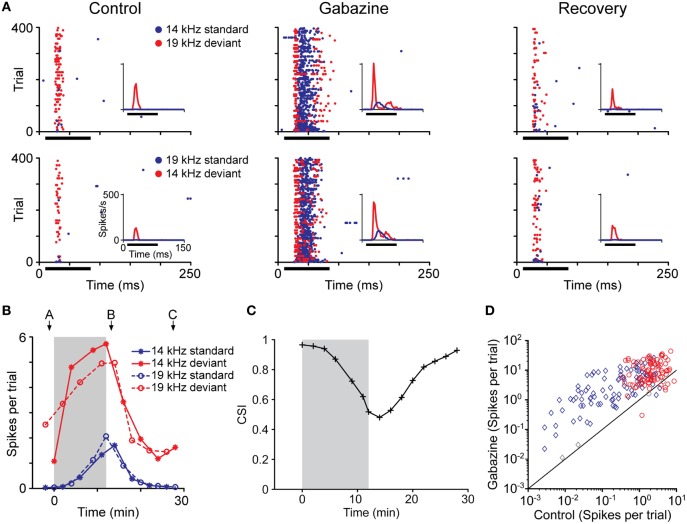
**Effect of the blockade of GABA_A_ receptors on the response magnitude for deviant and for standard stimuli. (A)** Dot rasters showing the effect of gabazine blockade of GABA_A_ receptors on the responses of a neuron with SSA. The frequencies tested in the oddball paradigm were 14 kHz and 19 kHz (Δ*f* = 0.3, 0.43 octaves). The insets show a PSTH of the response (3 ms bin size); the horizontal black bar indicates the duration of the stimulus. Before the application of gabazine, this neuron responded much more strongly to both frequencies when they were presented as the deviant than when they were presented as the standard stimulus, resulting in a CSI of 0.965. The application of gabazine for 12 min increased the response to both types of stimuli, but the relative increment was larger for the standards, causing the CSI to drop to 0.480. Fifteen minutes after the end of gabazine application, the neuron's response recovered to the control level, and the CSI increased to 0.926. **(B)** Evolution of the response magnitude of the neuron (mean spikes per trial) shown in **(A)** in response to standards (blue lines) and deviants stimulus (red lines). Note that the changes are similar for both frequencies (asterisk and circles) and the main differences are due to the probability condition. The shaded background represents the application of gabazine, which starts at *T* = 0. The arrows indicate the times corresponding to the dot rasters in **(A)**. **(C)** Evolution of CSI during the experiment. The symbols indicate the time at the end of a testing oddball sequence, so all times at or before 0 represent recordings completed before the start of the injection. **(D)** Effect of gabazine on response magnitude in the population of neurons. Gabazine increased the response (spikes per trial) of almost all neurons recorded, but the effect was different for standards (blue diamonds) than for deviants (red circles). Each symbol corresponds to one of the pair of stimuli for each neuron. Colored symbols indicate that the effect of gabazine was significant (Bootstrapping, 95% ci). Gray symbols represent changes that were not statistically significant. Reproduced from Pérez-González et al. (2012).

Recently, in a first attempt to study the role of GABAergic neurotransmission in the generation and/or modulation of SSA, gabazine an antagonist of GABA_A_ receptors was applied microiontophoretically, and the firing rate of neurons exhibiting SSA was recorded before, during and after the drug injection (Figures 10A–C; Pérez-González et al., 2012). The response magnitude (Figure 10D), discharge pattern and latency remained distinct for the deviant and standard stimuli. The main finding was that the blockade of GABA_A_ receptors modified the temporal dynamics of SSA but did not abolish it completely, although the CSI index was generally reduced. Adaptation to the standard stimulus still occurred in the absence of GABA_A_-mediated inhibition but it was slower, especially at the beginning of stimulation.

**Figure 11:**
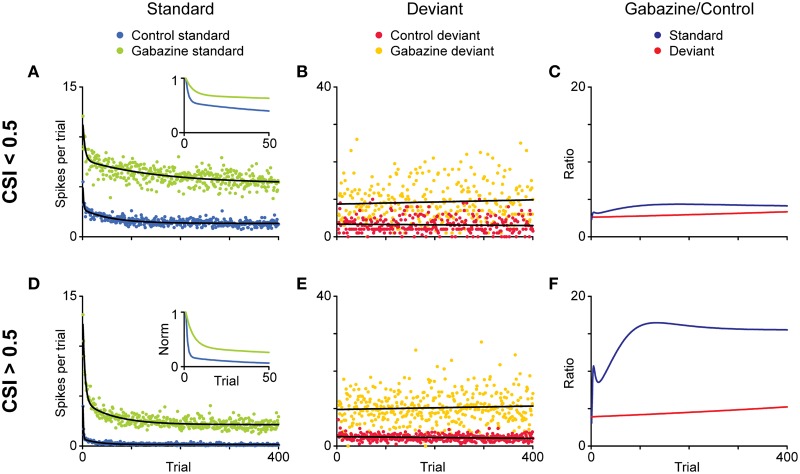
**Effect of the blockade of GABA_A_ receptors on the time course of adaptation and a model of SSA modulation by inhibition. (A,B; D,E)** Average discharge across the population of neurons for each position (trial) in the oddball sequence, separately for standard **(A,D)** and deviant **(B,E)** stimuli. The minimally adapting neurons (**A–C**; CSI < 0.5 in the control condition) and the highly adapting ones (**D,E**; CSI.0.5 in the control condition) were analyzed separately. Then the time course of habituation in the control condition and during application of gabazine was compared. The data for standard stimuli were fitted by a double exponential function (black lines), and the data for deviant stimuli, by a linear function. The insets show a magnified view of the normalized functions during the first 50 trials. Note the different ordinate scales for standard and deviant. **(C,F)** Ratio of gabazine/control for each type of stimulus during gabazine application. Reproduced from Pérez-González et al. (2012).

**Figure 12:**
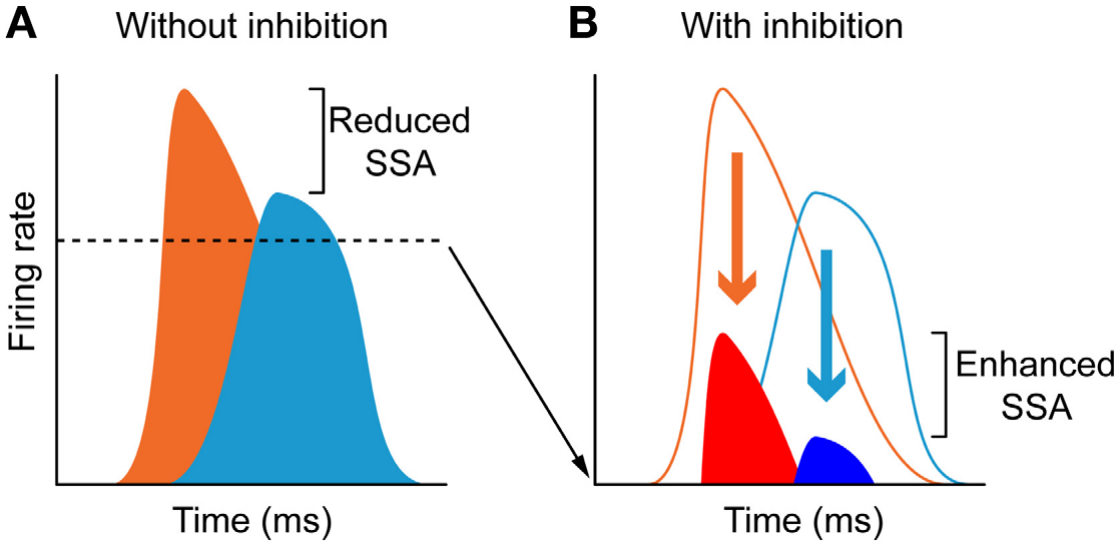
**The iceberg effect. (A)** Schematic representation of a model of the iceberg effect indicating that in the absence of inhibition, neurons respond to deviants (orange) and standards (light blue) with high firing rates, and thus the deviant to standard ratio is small. **(B)** Inhibition reduces the responses to both deviants (red) and standards (dark blue) increasing the deviant to standard ratio and thus enhancing SSA. The dashed line in **(A)**, delimit the amount of activity (bottom area of the histograms) that decreases due to inhibition. Reproduced from Pérez-González et al. (2012).

The time course of the adaptation to the standard stimulus has a rapid- and a slow-decay component, after which the response reaches a steady-state (Figures 11A–F). Both decay components are faster for the neurons exhibiting higher levels of SSA (CSI > 0.5) than for the neurons with less SSA (CSI < 0.5). The blockade of the GABA_A_ receptors slowed down both components and GABA inhibits more profoundly the steady-state component of the response of the neurons with the highest levels of SSA. As expected from previous results (Malmierca et al., 2009a; Zhao et al., 2011), Pérez-González et al. (2012) also found that the deviant-related activity did not decrease, showing a linear time course across the trials. The authors concluded that GABA_A_-mediated inhibition acts as a gain control mechanism that enhances SSA by controlling the neuron's gain and responsiveness. Thus, synaptic inhibition via GABA_A_ receptors seems to increase the saliency of the deviant stimulus (Figure 12). The role of inhibition in shaping contrast between stimuli has been described in detail in what is referred to as “the iceberg effect.” Briefly, the iceberg effect describes the observation whereby a neuron's spike output is more sharply tuned than the underlying membrane potential since only the strongest excitatory input sufficiently depolarizes the membrane to reach threshold for spike generation (Isaacson and Scanziani, 2011).

The results by Pérez-González and colleagues suggest that other factors must be involved in the generation of SSA, leading to an interest in exploring the role of GABA_B_ and glycinergic-mediated inhibition as well as neuromodulatory influences. GABA_B_ receptors may be key players in shaping SSA. These receptors seem to be involved in the processing of novel visual stimuli in the superior colliculus (Binns and Salt, 1997) and they regulate the release of glutamate from excitatory terminals in the DCIC through presynaptic mechanisms (Scanziani, 2000; Sun and Wu, 2009). Then, both pre- and postsynaptic mechanisms are likely to contribute to a reduction in the membrane depolarization after initial excitation. To dissect the interplay of synaptic excitation and inhibition is also necessary to understand the cellular and network mechanisms underlying SSA. Previously, Binns and Salt (1995) demonstrated that the activity mediated by the NMDA and AMPA/Kainate receptors modulates response habituation in the superior colliculus. Hitherto, no attempt has been made to determine whether SSA in single-unit activity in the IC depends on local glutamatergic neurotransmission.

Since the level of SSA varies within the neuronal frequency response area (Duque et al., 2012), it is likely that the mechanisms underlying SSA act at the sites of synaptic inputs on the IC neuron. The specific decrement to repetitive stimuli seen in neurons exhibiting SSA could be explained by adaptation occurring at frequency-segregated input channels (Eytan et al., 2003). The persistence of SSA at long ISI suggests that it is not merely a result of mechanisms such as synaptic fatigue (e.g., reduction in neuronal responses independently of the stimulus due to depletion of neurotransmitter vesicles). This is less likely to occur at very long interstimulus gaps. Delayed synaptic inhibition might account for the specific response suppression to a highly repetitive stimulus. This idea is supported by intracellular recording suggesting that excitation in neurons throughout the IC is often followed by long-lasting hyperpolarization, possibly due to synaptic inhibition (Covey et al., 1996; Syka et al., 2000; Wehr and Zador, 2005). This could also explain why the neurons with the highest SSA levels (Duque et al., 2012) exhibit onset response patterns to sound stimulation. Another possibility is short-term plasticity occurring at the sites of synaptic integration in IC neurons. These possibilities are not mutually exclusive and it could be that they operate in conjunction. It is plausible to think that the neuronal adaptation in the IC reflects high level network computations with strong participation of local inhibitory circuits (Pérez-González and Malmierca, 2012; Pérez-González et al., 2012), and with a modulatory control on SSA exerted by corticofugal projections (Anderson and Malmierca, 2013). Multidisciplinary efforts that will combine histological, electrophysiological, computational, and behavioral methods (Garagnani and Pulvermuller, 2011; Mill et al., 2011) will be necessary to unveil the basic mechanisms that generate SSA at each level along the auditory system.

## Concluding remarks and future directions

In this review, we have attempted to offer an account of the current state of SSA studies in the IC because of the growing interest on the single-neuron electrophysiology of auditory deviance detection. The dependence of neuronal SSA on various stimulus features, such as deviant probability and repetition rate, and the role of AC and of inhibition in shaping SSA at this auditory stage have been addressed.

Among the questions to be resolved in the study of auditory deviance detection are whether SSA is present along the entire auditory pathway or whether it is a regionalized phenomenon to certain structures and how the deviance coding is modified by bottom-up and top-down processes that take place at each station. The localization of the most strongly adapting neurons to frequencies in the non-lemniscal portions of the subcortical nuclei opens interesting question about the neuronal circuits involved since these parts of the auditory pathway process more complex acoustic features. To address these issues, it is necessary to record neurons in the brainstem nuclei (especially those that exhibit cross-frequency integration), to track the common input sources to neurons with strong SSA, as well as, their projections, and to make simultaneous recordings at two or more connected sites. Since stimuli in a natural scene vary in multiple features, another open question is whether neurons sensitive to frequency deviants are also deviance detectors for more complex sound patterns and whether the same neurons are capable of detecting deviant stimulus immersed in more complex forms of regularity (Cornella et al., 2012; Grimm and Escera, 2012). These and other questions await future studies.

### Conflict of interest statement

The authors declare that the research was conducted in the absence of any commercial or financial relationships that could be construed as a potential conflict of interest.
